# Monthly variations in self-report of time-specified and typical alcohol use: The Nord-Trøndelag Health Study (HUNT3)

**DOI:** 10.1186/s12889-015-1533-8

**Published:** 2015-02-21

**Authors:** Ann Kristin Knudsen, Jens Christoffer Skogen

**Affiliations:** Department of Health Registries, Norwegian Institute of Public Health, Kalfarveien 31, 5018 Bergen, Norway; Department of Global Public Health and Primary Care, University of Bergen, Kalfarveien 31, 5018 Bergen, Norway; Department of Public Mental Health, Norwegian Institute of Public Health, Kalfarveien 31, 5018 Bergen, Norway; Alcohol and Drug Research Western Norway, Stavanger University Hospital, Lagårdsveien 78, 4010 Stavanger, Norway

**Keywords:** Alcohol use, Seasonality, Epidemiology, Reliability, Validity, Self-report, Measurement error

## Abstract

**Background:**

Aggregated measures are often employed when prevalence, risk factors and consequences of alcohol use in the population are monitored. In order to avoid time-dependent bias in aggregated measures, reference periods which assess alcohol use over longer time-periods or measures assessing typical alcohol use are considered superior to reference periods assessing recent or current alcohol consumption. Alcohol consumption in the population is found to vary through the months of the year, but it is not known whether monthly variations in actual alcohol use affects self-reports of long-term or typical alcohol consumption. Using data from a large, population-based study with data-collection over two years, the aim of the present study was to examine whether self-reported measures of alcohol use with different reference periods fluctuated across the months of the year.

**Methods:**

Participants in the third wave of the Nord-Trøndelag Health Survey (HUNT3) answered questions regarding alcohol use in the last 4 weeks, weekly alcohol consumption last twelve months, typical weekly binge drinking and typical number of alcoholic drinks consumed in a 14 day period. For each of the alcohol measures, monthly variations in reporting were estimated and compared to the overall average.

**Results:**

Monthly variations in self-reported alcohol use were found across all alcohol measures regardless of reference period. A general tendency was found for highest level of alcohol use being reported during the summer season, however, the highest number of individuals who reported alcohol use in the last 4 weeks was found in January. Women reported substantially larger increase in weekly binge drinking during the summer months than men.

**Conclusions:**

Self-reports of alcohol use over longer time and typical alcohol use varies according to the month the respondents are assessed. Monthly variations should therefore be taken into account when designing, analyzing and interpreting data from population-based studies aimed to examine descriptive and analytical characteristics of alcohol use in the population.

## Background

Hazardous alcohol use constitutes a considerable burden for the society [1,2]. Reliable and valid measures are pivotal in order to monitor alcohol use in the population and guide intervention strategies. Monitoring of alcohol consumption is primarily based on two main sources of information: numbers derived from statistics on alcohol production, sales and taxation; and self-report data on alcohol use from epidemiological studies [3]. The main aims in many studies based on self-reported alcohol consumption are to establish levels of risky alcohol consumption and total volume of alcohol consumed over time in the population (see i.e. [4-7]). The responses on specific alcohol measures are therefore often aggregated to annual consumption or typical consumption estimates to get an overview of the population’s drinking habits [8]. Aggregated alcohol measures are also often employed when prevalence, risk factors and consequences of alcohol use in the population are investigated (see i.e. [7,9-11]. For self-report data, the framing of the time period in which the respondents are asked to report their alcohol use (the reference period) is of importance. These reference periods vary widely between studies, ranging from the day before the assessment to the last 12 months. Detailed questions on alcohol use with a short reference period (i.e. diary methods or questions about yesterday’s alcohol consumption) are found to give data that are closer to actual consumption in the time-period of interest than questions about ”typical” alcohol consumption [4,12,13]. However, detailed questions with short time-frame do not capture longer-term drinking patterns [4], and they also tend to miss out on sporadic heavy drinking occasions, such as holidays and festivals (i.e. Christmas, Easter or the summer holidays), as data-collection usually are conducted outside these periods. The risk of adverse outcomes of alcohol use increase with episodes of heavy drinking and with prolonged drinking periods [1,2,8], and thus these alcohol use patterns may be of particular importance to alcohol researchers interested in the hazardous and long-term effect of alcohol use. Reference periods covering several months, or questions not using reference periods at all, are thought to eliminate time-dependent bias such as atypical drinking events or fluctuations, such as seasonality in alcohol consumption, and give more representative average values of quantity and frequency of drinking [14]. The most common methods used to assess long-term or typical alcohol consumption habits are the quantity-frequency method (QF) and the graduated frequency method (GF) [4,12,15-17], which asks the respondents to indicate how often they typically or in a given, usually prolonged, time-period have a drinking occasion, and the quantity they usually consume on these occasions. In some extensively used alcohol screening instruments, such as the Alcohol Use Disorders Identification Test (AUDIT) [18,19], the questions are framed without a specified reference period and are thus assumed to measure the respondent’s typical alcohol use.

Responders seem to employ different cognitive strategies when they respond to questions regarding their typical alcohol consumption or their past consumption over time [20], and they tend to remember recent drinking events more vividly than older events [21]. Reporting of typical alcohol consumption may therefore be unduly influenced by the respondent’s recent drinking behavior [22]. For instance has reporting of alcohol consumption been found to be clustered around the day the interview took place [14,21]. Thus, time-dependent bias in reporting of alcohol consumption may be present even if the questions are framed in terms of long-term or typical alcohol habits.

As alcohol is commonplace in many annual celebrations and activities, alcohol use varies with the annual seasons. “Seasonality in alcohol consumption” has been observed in alcohol research since the mid-1960s [23], and previous studies have shown a peak of number of persons reporting to be using alcohol in December [24-27], with consumption found to be 70% higher the last two weeks of December than “normal weeks” of the year [25]. Excluding December, it is less clear whether alcohol use varies across the months of the year, and observations of seasonality seems to differ according to the temperature, latitude and culture in the countries the studies are conducted. A tendency of more people reporting alcohol use in the summer compared to the winter months, both in terms of frequency of drinking episodes and number of alcohol units consumed per episode, has been found in the United States, the Netherlands, Scotland and Estonia [23-29], but not in Spain [30]. One study found seasonal variation in alcohol abstention, in which 28% of female self-defined abstainers reported that they drank alcohol during Christmas [25]. In general, similar patterns are found for seasonal variations in alcohol use for men and women [24,25,27], except for seasonality in heavy drinking, which has been found to be more pronounced for women compared to men, and especially expressed through more women engaging in binge drinking of wine during spring and summer [25]. Men, in contrast, are found to have more stable heavy drinking patterns, and tend to prefer beer throughout the year [25].

Due to the tendency of respondents to remember and therefore base their reporting on alcohol consumption on their recent alcohol use, seasonal variations in alcohol use may affect responses on long-term or typical alcohol use, which again may affect the validity of such data as sources of information on risky alcohol consumption and total volume of alcohol consumed over time in the population. However, this potential source of measurement bias has been scarcely examined. Two previous studies, published in 1978 and 1992 [23,31], have done some investigation into whether there is seasonality also in the reporting of more typical alcohol consumption. Fitzgerald and colleagues noted that responders reporting how often they usually had a drink and how many drinks they ordinarily consumed during a 12 month period differed according to whether they were asked in February and March, or in June through September, with higher reporting in the summer months [23]. Further, Midanik has described how responders tended to report lower alcohol consumption in the last 12 months if they were asked in January compared to December [31]. Although informative, the data from these studies were taken from small samples and may not be generalizable to a modern day context. It is therefore a need for updated knowledge, based on epidemiological data, on whether the reporting of prolonged or typical alcohol use differs due to the month the assessment is done.

Using a large epidemiological study, in which data-collection was conducted over a two-year period, the main objective with the present study was to investigate whether reporting of alcohol consumption differed according to the month the assessment was conducted. Furthermore, we aimed to examine whether a monthly variation in reporting of alcohol consumption differed according to the framing of the reference period, namely whether a monthly variation could be found regardless of whether the reference period indicated a specified time-frame (the last 4 weeks and the last 12 months), or whether the respondents were asked to report on their typical alcohol consumption, with no specified reference period indicated.

## Methods

### Participants and measures

The study population consisted of participants in the third wave of the population-based Nord-Trøndelag Health Survey (HUNT3), conducted between October 2006 and June 2008 in the county of Nord-Trøndelag in Norway [32]. Data-collection included a general physical examination, sampling of blood and urine, and several questionnaires. All participants with valid information on date of participation, and valid responses on one or more questions regarding alcohol consumption (N = 50,814) from questionnaire 1 (Q1) were eligible for the present study.

Q1 was included with the invitation letter for survey participation, and was returned by the participants when they attended the health examination sites [32]. Date of participation in HUNT3 was available for all who agreed to participate, and was recoded from exact dates to month of participation for the purposes of this study. Due to differences in data-collection intensity, the number of responses each calendar month ranged from N = 6,107 in January to N = 2,261 in December, with the exception of July where only N = 269 responders participated.

Several questions regarding alcohol consumption were included in Q1, and the framing of reference period varied between the questions. Two questions had a specified reference period, namely the last 12 months and the last 4 weeks. Frequency of alcohol consumption was assessed for the previous year, with question framed as *“About how often in the last 12 months did you drink alcohol?”*, with eight different response categories “4-7 times a week”, 2–3 times a week”, “about once a week”, “2-3 times a month”, “about once a month”, “a few times a year”, “not at all the last year”, “never drink alcohol”. These responses were recoded into a binary variable of weekly alcohol consumption (yes/no) during the last 12 months, in which the three first original categories indicated that participants reported weekly consumption, and the latter five indicated less than weekly consumption. The frequency question was then followed by one question regarding alcohol use the last four weeks: *“Did you drink alcohol during the last 4 weeks?”* (yes/no). Then followed two questions where the participants were asked to report on their typical alcohol use, thus no reference period was given. Typical consumption of different types of beverages during a 14-day period was assessed with the question *“How many glasses of beer, wine or spirits do you usually drink in the course of two weeks”*, in which the participants should fill in the “number of glasses” of beer, wine and spirits in three separate boxes. The responses yielded three different variables, indicating typical number of glasses of beer, wine and spirits drunk during a 14-day period. Finally, the participants were asked about their typical binge drinking habits. The participants were asked *“How often do you drink 5 glasses or more of beer, wine or spirits in one sitting?”*, with the following response categories “never”, “monthly”, “weekly” and “daily”. The responses were recoded into a binary variable of “weekly binge drinking” (yes/no), were ticking off “weekly” or “daily” indicated “yes” and the other categories indicated “no”. The total number of alcohol-variables included in the present analyses was six.

We also included information about the respondent’s age and gender, which was retrieved from the Norwegian Tax Administration.

### Statistical procedure

To retain maximum number of respondents, we included all individuals with valid responses for each alcohol-variable in separate analyses. The number of responses from the pool of N = 50,814 eligible participants therefore differed for each analysis (ranging from n = 34,032 to n = 49,451). For the variables related to typical consumption of beer, wine and spirits during a 14-day period, the mean number of glasses was computed for each month of the year. For the variables assessing any alcohol consumption last 4 weeks (yes/no), weekly alcohol consumption last 12 months (yes/no) and typical weekly binge drinking (yes/no), the proportion of monthly positive responses was calculated. In order to compare the reported typical consumption of beer, wine and spirits across months of the year, we employed negative binomial regression modelling (NBR) for over-dispersed variables and many data points at zero [33,34]. For all three beverages, the likelihood ratio test comparing NBR with Poisson regression models was p > 0.0001, strongly suggesting that NBR modelling was more appropriate than Poisson regression modelling [35]. The reported beverage-specific contrasts express the difference between number of predicted glasses consumed for each month relative to the overall reported mean number of glasses consumed. Logistic regression analysis was employed to examine reported alcohol consumption last 4 weeks, weekly alcohol consumption last 12 months and typical weekly binge drinking across the months of the year. In concordance with previous research [26,27], we used «deviation from means» coding as the reference category [36]. The reported odds ratios therefore reflect the odds of reporting the drinking behaviour in question for that month (month-specific logit) compared to the overall odds (average logit for all months). As an external validity check to self-report data obtained from the HUNT3, we also present the registered mean monthly sale of alcoholic beverages (beer, wine and spirits) in Norway for the years 2006 and 2007. This data was obtained from Vinmonopolet (The Norwegian Wine and Spirits Monopoly, which have exclusive right to retail wine, spirits and strong beer in Norway) and the Norwegian Beer and Soft Drinks Producers association. All analyses were computed for the complete sample and later stratified by gender. Stata 13.1 for Windows was used for all analyses [37]. Unless otherwise stated, the monthly variation for the included alcohol measures given in the results section was statistically significant (at least p < 0.05).

### Ethics

The HUNT3 survey was approved by the Regional Committee for Ethics in Medical Research (REK), and the Norwegian Data Inspectorate. Written informed consent was obtained from all subjects. The present study was approved by REK Western Norway.

## Results

**Figure 1:**
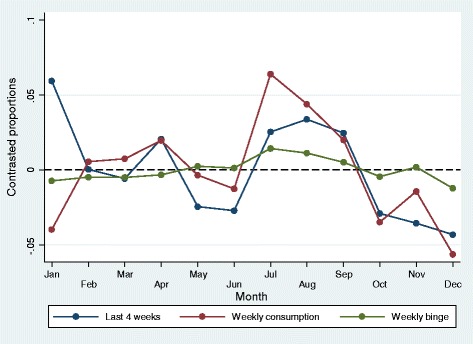
**Predicted probability of any alcohol consumption last 4 weeks (N = 49,031), weekly alcohol consumption last 12 months (N = 49,451) and weekly binge drinking (N = 47,606) across months of the year.** Compared to overall proportion.

The mean age of all the HUNT3 participants was 53.1 (standard deviation 16.1) ranging from 19 to 100 years, and 54.6% of the participants were female. All of the alcohol measures based on self-report in HUNT3 fluctuated to some degree according to the month of year the assessment was conducted (See Table 1 and 2). The highest number of persons who reported to have consumed alcohol in the last 4 weeks was found in January (Table 1 and Figure 1). Compared to the overall odds, the odds ratio (OR) for reporting consumption the last 4 weeks was 1.42 in January, with increased odds also for April, August and September, and decreased odds for May, June, October, November and December (all p-values at least <0.01, see Table 1). For reported weekly consumption the last 12 months, April, July, August and September had increased odds, while January, October and December had decreased odds (all p-values at least <0.05, see Table 1). Reported typical weekly binge drinking had increased odds for August, and decreased odds for January and December (p-values at least <0.05, Table 1).

**Table 1:** **Proportion of HUNT participants who report any alcohol consumption last 4 weeks, weekly alcohol consumption last 12 months and typical weekly binge drinking across the months of the year**

	**Any alcohol consumption last 4 weeks (N = 49,031)**		**Weekly alcohol consumption last 12 months (N = 49,451)**		**Typical weekly binge drinking (N = 47,606)**	
	**Proportion**	**Odds ratio (OR)**	**Proportion**	**OR**	**Proportion**	**OR**
Overall average	0.758 [0.754,0.762]	Ref	0.368 [0.364,0.373]	Ref	0.034 [0.032,0.036]	Ref
January	0.818	1.42***	0.334	0.84***	0.028	0.81**
	[0.808,0.828]		[0.322,0.346]		[0.024,0.033]	
February	0.759	0.99	0.379	1.03	0.031	0.88
	[0.748,0.770]		[0.367,0.392]		[0.026,0.035]	
March	0.753	0.96	0.381	1.04	0.031	0.87
	[0.742,0.765]		[0.368,0.394]		[0.026,0.035]	
April	0.779	1.11**	0.394	1.09**	0.033	0.93
	[0.768,0.790]		[0.381,0.406]		[0.028,0.037]	
May	0.734	0.87***	0.370	0.99	0.038	1.10
	[0.722,0.746]		[0.358,0.383]		[0.033,0.043]	
June	0.732	0.86***	0.361	0.95	0.037	1.06
	[0.718,0.745]		[0.347,0.376]		[0.031,0.043]	
July	0.784	1.15	0.438	1.31*	0.050	1.45
	[0.734,0.834]		[0.378,0.498]		[0.023,0.077]	
August	0.793	1.21***	0.418	1.21***	0.047	1.36***
	[0.779,0.807]		[0.401,0.435]		[0.040,0.054]	
September	0.783	1.14**	0.394	1.09*	0.041	1.18
	[0.766,0.800]		[0.374,0.414]		[0.033,0.049]	
October	0.730	0.85***	0.339	0.86***	0.031	0.89
	[0.717,0.743]		[0.325,0.353]		[0.026,0.036]	
November	0.723	0.82***	0.360	0.94	0.038	1.08
	[0.710,0.736]		[0.346,0.373]		[0.032,0.043]	
December	0.716	0.79***	0.318	0.78***	0.024	0.67**
	[0.697,0.735]		[0.298,0.337]		[0.017,0.030]	

*p < 0.05, **p < 0.01, ***p < 0.001.

Odds ratios reflect odds relative to overall average odds.

**Table 2:** **Mean number of glasses of beer, wine and spirits typically consumed within 14 days across months of the year**

	**Glasses of beer (N = 38,248)**		**Glasses of wine (N = 40,136)**		**Glasses of spirits (N = 34,032)**	
	**Mean**	**Contrast**	**Mean**	**Contrast**	**Mean**	**Contrast**
Overall average	2.20 [2.16,2.23]	Ref	2.39 [2.35,2.42]	Ref	1.04 [1.02,1.06]	Ref
January	2.06	-.23***	2.09	-.27***	1.02	-.02
	[1.96,2.15]		[2.01,2.18]		[0.95,1.08]	
February	2.08	-.21***	2.44	.08	1.11	.07
	[1.98,2.17]		[2.35,2.54]		[1.04,1.17]	
March	2.15	-.14**	2.51	.15**	1.01	-.03
	[2.05,2.24]		[2.41,2.61]		[0.94,1.08]	
April	2.15	-.14*	2.47	.11*	1.03	-.01
	[2.06,2.25]		[2.38,2.57]		[0.97,1.10]	
May	2.18	-.11	2.63	.27***	1.11	.07*
	[2.04,2.32]		[2.52,2.75]		[1.04,1.19]	
June	2.27	-.02	2.42	.05	1.06	.02
	[2.14,2.39]		[2.30,2.53]		[0.97,1.15]	
July	2.94	.66*	2.23	-.13	1.11	.07
	[2.23,3.66]		[1.86,2.61]		[0.84,1.39]	
August	2.58	.29***	2.91	.55***	1.07	.03
	[2.42,2.74]		[2.76,3.06]		[0.98,1.17]	
September	2.60	.31***	2.52	.16*	0.94	-.10*
	[2.38,2.81]		[2.35,2.69]		[0.84,1.04]	
October	2.12	-.17**	2.05	-.31***	1.01	-.03
	[2.00,2.24]		[1.95,2.14]		[0.93,1.09]	
November	2.15	-.14*	2.28	-.08	1.01	-.03
	[2.04,2.25]		[2.18,2.39]		[0.94,1.08]	
December	2.19	-.10	1.78	-.58***	1.00	-.04
	[2.02,2.35]		[1.65,1.92]		[0.90,1.09]	

*p < 0.05, **p < 0.01, ***p < 0.001.

Compared to grand mean using negative binomial regression.

**Figure 2:**
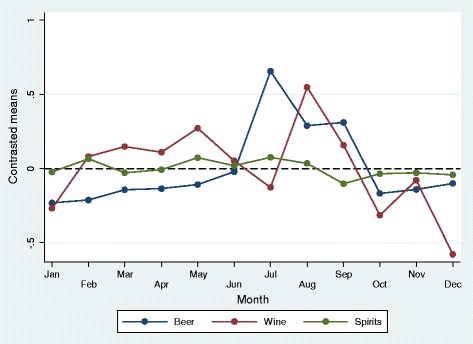
**Predicted number of glasses typically consumed within 14 days for beer (N = 38,248), wine (N = 40,136) and spirits (N = 34,032) across months of the year.** Compared to grand mean.

For self-reported typical consumption of beer, wine and spirits, the number of reported glasses of beer compared to the grand mean was higher in July-September, and lower in January-March, October and November (p-values at least <0.05, Table 2). The number of glasses of wine typically consumed was higher in March-May, August and September, and lower in January, October and December (p-values at least <0.05, Table 2). Compared to beer and wine, there was much less monthly variations in the reporting of glasses of spirits typically consumed, and only May was significantly higher than the grand mean, while September was lower (both p < 0.05, Table 2). The monthly variations in typical beverage specific consumption are visualized in Figure 2.

**Figure 3:**
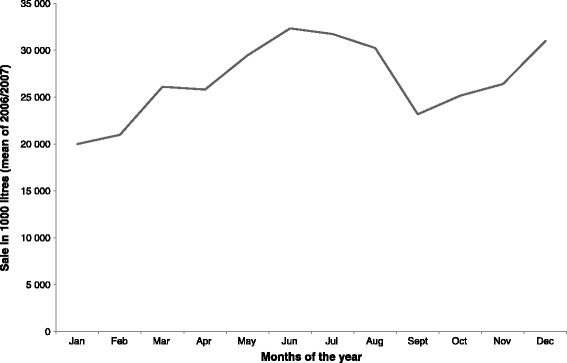
**Total registered alcohol sale in Norway across months of year (monthly mean of 2006 and 2007.**

In accordance with official Norwegian alcohol sale numbers across the year (Figure 3), the highest levels of typical alcohol consumption were reported when use was assessed during the spring and summer season (Table 1 and Table 2, and Figure 1 and Figure 2). However, while alcohol sale also peaked in December, higher level of self-reported alcohol consumption in December was only found for the measure of alcohol consumption in the last 4 weeks when assessed in January in HUNT3.

For the variables measuring typical consumption of different types of beverages during a 14-day period, the highest difference from the grand mean was found in July for beer, while typical consumption of wine peaked in August (Table 2 and Figure 2). Self-report of any alcohol consumption during last 4 weeks differed most from January to December. Weekly alcohol consumption last twelve months and weekly binge drinking peaked in July and August (Table 1 and Figure 1).

The overall patterns in the gender-stratified analyses were similar for men and women for all alcohol variables, showing a peak during the summer months and lower levels during the other months of the year (data not shown). There was, however, one notable difference in level of fluctuation for women compared to men in relation to weekly binge drinking; for which women had a OR of 2.32 (p = 0.045) relative difference from the overall gender-specific odds, compared to a OR of 1.25 (p = 0.510) for men. As there were fewer responses in July, we also investigated the odds ratio for the summer period (June-August), and found the same pattern of increased reported weekly binge drinking for women (OR 1.37 (p < 0.001)) compared to OR 1.18 (p = 0.004) for men.

## Discussion

In the present study, based on a large sample from the general population assessed over a two-year period, we found considerable monthly variations in reporting across a range of alcohol measures. A general tendency was found for highest level of alcohol consumption being reported in the summer months, and this corresponded with the registered sales data from Norwegian alcohol retail. The tendency was found regardless of reference period, with one notable exception: the highest number of individuals who reported alcohol use in the last 4 weeks was found in January. Overall, the monthly variations in self-reported alcohol use were similar for men and women, except that women reported substantially larger increase in weekly binge drinking during the summer months than did men.

### Monthly variations in reporting of recent alcohol use

Studies from European countries and the United States have found a tendency of alcohol use to peak in December and during the summer months [23-29]. The reporting of recent alcohol use (last 4 weeks) in the present study are in line with these results, as our results indicates equal to or higher effects of seasonal variations compared to previous studies. For example, in the present study the odds ratio of reporting any alcohol consumption last 4 weeks in January was 1.42 while two previous studies reported odds ratios of 1.17 [27] and 1.22 [26]. The highest number who reported alcohol use in the last 4 weeks in the HUNT3 data was found in January, when over 80% of the sample reported that they had consumed alcohol the previous month. There was also an increased odds of reporting alcohol use in the last 4 when asked in April, August and September – reflecting increased alcohol use during Easter and in the Summer months. Thus, there is likely to be seasonal variations in alcohol use also in Norway.

### Monthly variations in reporting of alcohol use the last 12 months and typical alcohol use

In contrast to the findings on recent alcohol use, when people were asked to report on whether they used alcohol weekly in the last 12 months, or whether they engaged in typical weekly binge drinking, the number of people answering affirmative on these questions were found to be lowest in January and highest in the summer months. These findings are indicating that monthly variations in actual alcohol consumption in some degree affect the reporting of more long-term alcohol consumption. The lower reporting in January is somewhat contrary to the hypothesis that reporting on alcohol use is based on recall of recent drinking behavior, but is in line with a previous study which showed that reports of drinking in the past 12 months were lower when assessed in January compared to December [31]. These findings could perhaps be explained by people considering their alcohol use during Christmas as atypical, and thus leaves the Christmas indulgence out of the equation when asked to estimate their typical alcohol use. This may not be the case during the summer months, when people may have engaged in higher levels of alcohol use over an extended period of time. As a consequence, the behavior may not stand out in their mind as atypical or exceptional.

Further, in contrast to the alcohol sale numbers, the level of alcohol use last 12 months (weekly drinking) and typical alcohol use (weekly binge drinking and number of alcoholic beverages) was also reported to be lower when this was assessed in December. An explanation for this finding may be that HUNT3 data collection in December was conducted during the first three weeks of the month, and thus before the Christmas festive season began.

In relation to binge drinking, the pattern of more monthly variation for women compared with men was also found in a previous study [25]. This finding could indicate that female binge-drinking is more situation-specific, while male drinking is more habitual [25]. If so, self-reported binge drinking among men may be less prone to time-related bias than among women. Regardless, it should be noted that monthly variations in self-reported binge drinking was present for men as well.

### Implications of the results

The major difference between the present study and previous studies in this area is that while the previous studies employed defined reference periods, ranging from the current day [25], or previous week [25], two-weeks [30], month [26-28] or 6 months [25] before the assessment, the present study also included questions on *typical* alcohol use, without any specific reference period. Thus, while previous studies have primarily focused on assessing seasonal variations in alcohol use per se, the present study also examined whether the anticipated seasonal variations in alcohol use may affect reporting on measures assumed to minimize or eliminate time-dependent bias, such as QF or GF measures [4,12,15-17]. In 2005, Heeb and Gmel claimed that short reference periods “may be plagued with errors related to clustering effects of the day of the interview” (p.1015 [14]). Longer reference periods are generally believed to reduce the time-related effects associated with atypical drinking episodes and variations in alcohol consumption patterns affected by cultural or seasonal aspects, and thus obtain more representative average values of long-term and stable patterns of alcohol use. Our results indicate that this may not be the case, and that regardless of reference period, self-reported measures tend to assess current or recent rather than typical alcohol use in the population. This notion is further strengthened by the registered sales data obtained from external sources. Reports of alcohol use seem to fluctuate between the months of the year even when the respondents are asked about their alcohol use the last 4 weeks, the last 12 months, or their typical alcohol use.

The reliability of the reporting of typical or 12 months alcohol use depends on both the respondents’ ability to remember past drinking behavior, and on their ability to estimate the average use across this time span. This is a rather demanding task, and the memory of recent alcohol use episodes may stand out more vividly than more distant ones, and affect the reporting. The monthly variations in reporting of long-term and typical alcohol use is thus probably due to responders giving answers based on their recent alcohol habits, as these comes easier to mind than more distant or typical alcohol use [20,21]. Thus, responders tend to be quite imprecise in their estimates of alcohol consumption when a longer or an unspecified time-frame is introduced [23].

Previous studies that have examined the issue of seasonality in alcohol consumption conclude that studies that do not take seasonal variation into account when planning the study or interpreting the results may be biased [23-30]. Monthly variations in reported alcohol use are likely to produce bias in studies that aims to estimate the general incidence and prevalence of alcohol use in the population, and in studies that employ aggregated estimates of alcohol use. As an example from our analyses the mean prevalence of weekly binge drinking during the winter season (December, January and February) is 2.9% versus 4.2% during the summer months (June, July and August). Depending on the month or season the data is collected, the estimate generated may thus be either an over-estimate or an under-estimate of the average alcohol use during a year. Monthly variations in reporting of alcohol use may also have an impact of effect estimates, For instance, people who report weekly binge drinking behavior in a month where this is relatively rare in the population (i.e. January) may differ from people who report weekly binge drinking in a month where this is more common (i.e. August). Thus, a person who may be considered a case in August may be a control in January, and this may affect the effect estimates.

Already in 1978, Fitzgerald and colleagues recommended caution when data based on measures beyond time-specific estimates of consumption, for instance projections of annual consumption or trends in annual consumption, were interpreted [23]. The validity of aggregated data on alcohol consumption based on self-reports may be particularly biased in studies with short data-collection frames, and caution is required when data on alcohol consumption collected in a short time-frame are compared with other data. Thus Fitzgerald further suggested that “interviews should be conducted at comparable times of the year” (p. 883 [23]). Other authors have also given support to this recommendation, for instance concluded Lemmens and Knibbe that “comparisons of survey data on drinking are more or less invalid if the respective time-frames do not correspond” [25].

Our results indicate that even though both the time and the geographic location are different in our study compared to the previous studies, and even though we mainly focused on reports of typical alcohol use or alcohol use in the last 12 months, these concerns are still relevant. Framing of questions of alcohol consumption with the use of prolonged or typical reference periods do not seem to eliminate seasonal variations in reporting of alcohol consumption.

### Strengths and limitations

The present study holds several strengths: Firstly, the data comes from a large well-defined population-based study, ensuring a sufficient sample size to investigate monthly variations in self-reported alcohol consumption as well as potential gender-differences. Secondly, the data collection had a time-frame of almost 2 years (ranging from October 2006 to June 2008) making it less likely that any monthly variation was due to some anomaly for a given calendar year. Thirdly, the present study included several different measures of alcohol use, with both specified and un-specified reference periods. As a result we were able to investigate the potential monthly variation in self-reported alcohol use across different types of question formulations with different reference periods. There is now general agreement that one should include questions on both frequency of drinking events, quantity of alcohol units consumed per drinking event and more irregular episodes of heavy drinking when alcohol consumption is assessed [4,12,15-17], and all these measures were included in the present study.

Despite these strengths, the study also holds some notable limitations: Firstly, when considering generalizability of the results, the geographical location of the study setting should be taken into account. The county of Nord-Trøndelag consists of mainly rural areas, and is located in Mid-Norway. The high latitude of the setting means that there are large differences in hours of sunlight and temperature between summer and winter [38]. These contextual factors may have an impact on both actual alcohol consumption and on self-report patterns, although the official sales statistics of Vinmonopolet shows a similar monthly variation in Nord-Trøndelag as for Norway as a whole for the 2006 and 2007 (data not shown). Previous studies employing wave two of the HUNT-study (HUNT2) have not found any seasonal variation in self-reported insomnia, time in bed or prevalence of case-level anxiety as measured by the Hospital Anxiety and Depression Scale (HADS) [39,40]. A seasonal variation for prevalence of case-level depression has, however, been reported [39]. There is thus little reason to believe that our findings are reflections of a general seasonal variation in self-report patterns, but rather that they either reflect variations in actual alcohol habits during the year or self-report patterns specifically related to questions regarding alcohol consumption. Secondly, a limited number of individuals participated in July, and the results from this month must therefore be interpreted with caution. Despite this, the patterns seen in July were in accordance with the other summer months. Thirdly, although several different questions regarding alcohol consumption were included, it would be interesting to compare the monthly variations seen in this study with other techniques of data collection on alcohol consumption through self-report, such as a records in an alcohol diary, or measures of more harmful alcohol use patterns, such as problem drinking and alcohol use disorders. Fourthly, although beyond the scope of the present paper, it would have been advantageous to assess the impact the monthly variations observed with respect to an outcome from an external data source, such as life-time clinically diagnosed alcohol use disorder. This would have enabled an investigation of whether seasonal variation in the reporting of alcohol use leads to a differential association with important outcomes. Fifthly, the information obtained regarding registered sales does not include tax-free sales and home-produced alcoholic beverages. There is, however, little reason to assume that the lack of such information invalidates the pattern shown in Figure 3. Finally, nonparticipation is always a challenge in epidemiological studies, and although data from HUNT3 in general are considered representative for the general population of Norway, selection bias cannot be excluded [32]. In particular, nonparticipation in health surveys is found to be higher among individuals with alcohol problems [41,42]. It is thus likely that individuals with a high and stable alcohol consumption pattern did not participate in the present study.

## Conclusion

Monthly variations in self-report measures of alcohol use may affect both general estimates of alcohol consumption and prevalence estimates of at-risk drinking, and effect estimates between alcohol consumption and various risk factors and consequences. Monthly variations should therefore be taken into account when designing epidemiological studies aimed to assess alcohol use in the population. The data-collection should be distributed throughout the year, and monthly variations in alcohol use should be taken into account when data is analyzed and interpreted.

## Abbreviations

AUDIT: The Alcohol Use Disorders Identification Test
GF: Graduated frequency method
HADS: The Hospital Anxiety and Depression Scale
HUNT: The Nord-Trøndelag Health Survey
NBR: Negative binomial regression modelling
OR: Odds ratio
REK: Regional Committee for Ethics in Medical Research
QF: Quantity-frequency method (QF)
